# Systemic microbial TLR2 agonists induce neurodegeneration in Alzheimer’s disease mice

**DOI:** 10.1186/s12974-020-01738-z

**Published:** 2020-02-14

**Authors:** Neta Lax, Nina Fainstein, Yossi Nishri, Ayal Ben-Zvi, Tamir Ben-Hur

**Affiliations:** 1 Department of Neurology, The Agnes Ginges Center for Human Neurogenetics, Hadassah – Hebrew University Medical Center, Jerusalem, Israel; 2 Department of Developmental Biology and Cancer Research, Institute of Medical Research Israel-Canada, Hebrew University - Hadassah Medical School, Jerusalem, Israel

**Keywords:** Alzheimer, 5xFAD, Neurodegeneration, Microglia, TLR2

## Abstract

**Background:**

Accumulating data suggest a central role for brain microglia in mediating cortical neuronal death in Alzheimer’s disease (AD), and for Toll-like receptor 2 (TLR2) in their toxic activation. Amyloid deposition in preclinical AD is associated with microglial activation but not directly with neurodegeneration. We examined in transgenic 5xFAD mice the hypothesis that systemic TLR2 agonists, derived from common infectious agents, may accelerate neurodegeneration in AD.

**Methods:**

Microbial wall-derived TLR2 agonists zymosan and lipoteichoic acid were administered intraperitoneally or intracerebroventricularly to 7-month-old wild-type or 5xFAD mice. Immunofluorescent stainings were used to quantify cortical neurons and evaluate tissue reaction. Microglial activation was assessed using functional assays, RNA expression, and FACS analysis.

**Results:**

Repeated low-dose systemic administration of zymosan or lipoteichoic acid killed cortical neurons in 5xFAD mice but not in wild-type mice. Direct CNS delivery of a selective TLR2 antagonist blocked the neurotoxicity of systemically administered zymosan, indicating that CNS TLR2 mediates this effect. Systemically administered zymosan crossed the disrupted blood-brain barrier in 5xFAD mice and entered brain parenchyma. By intracerebroventricular delivery, we found a dose- and exposure time-dependent acute neurotoxic effect of the microbial TLR2 agonist, killing cortical neurons. 5xFAD mice exhibited significantly increased vulnerability to TLR2 agonist-induced neuronal loss as compared to wild-type mice. Microbial TLR2-induced neurodegeneration was abolished by inhibiting microglia. The vulnerability of 5xFAD mice brains was mediated by an increase in number and neurotoxic phenotype of TLR2-expressing microglia.

**Conclusions:**

We suggest that repeated exposure to microbial TLR2 agonists may facilitate neurodegeneration in AD by their microglial-mediated toxicity to the hyper-vulnerable environment of the AD brain.

## Introduction

Accumulating evidence suggests that the neurodegenerative process in AD is driven by the neurotoxic effect of activated microglia [20, 37]. Microglia, like other monocyte-derived cells, express a range of Toll-like receptors (TLRs), and may be activated by pathogen-associated molecular patterns (PAMPs) and pro-inflammatory cytokines reaching the CNS (central nervous system) parenchyma. Microglial TLR2 plays a role in mediating pathological processes in AD. TLR2 serves as a receptor for Amyloid beta (Aβ)-induced microglial activation [21]. TLR2 mediates Aβ ingestion by microglia, and its blockage results in amyloid accumulation [23]. Furthermore, disruption of downstream TLR2 signaling prevented the progression of AD pathology in AD transgenic mice [30]. However, Aβ deposition is probably not associated directly with neurodegeneration [6, 12], suggesting that other superimposing insults, such as external TLR2 agonists, may be involved in the progressive loss of cortical neurons.

The bacterial wall of multiple types of microorganisms causing common infections contains molecules that bind and activate TLR2. Prolonged worsening of cognitive function was observed long after the resolution of a systemic infection in AD patients ([9, 15]. It has been suggested that systemic infections might activate inflammatory cells in the brain and especially microglia to exacerbate disease ([14, 28]. Indeed, a role of infectious organisms facilitating the rate of disease progression has been suggested [29] and further supported by recent literature linking specific infectious agents with AD progression ([11, 43].

We hypothesized that microbial TLR2 agonists may induce neurodegeneration by a direct effect on the brain. Here, we examined in 5xFAD mice, a transgenic model of AD, whether microbial TLR2 agonists induce neurodegeneration and whether the Alzheimer pathology renders the brain more vulnerable to the neurotoxic consequences of microbial TLR2 agonists. We found that repeated systemic injections of the microbial wall TLR2 agonists zymosan and lipoteichoic acid (LTA) kill cortical neurons in 5xFAD mice, at a time point of marked AD pathology, but before spontaneous neuronal loss. Direct intracerebroventricular (ICV) delivery of a selective TLR2 antagonist blocked the neurotoxicity of systemically administered zymosan. The disrupted blood-brain barrier in 5xFAD mice enabled the penetration of the TLR2 agonist into the CNS. Direct intracerebroventricular (ICV) delivery of the microbial TLR2 agonist showed a dose- and exposure time-dependent neurotoxic effect, and a significantly increased vulnerability of AD mice to the TLR2 agonist as compared with wild-type mice. Microbial TLR2–induced neurodegeneration was mediated by an increase in number and neurotoxicity of activated microglia. In conclusion, we suggest that repeated infections, with exposure to microbial TLR2 agonists, may facilitate neurodegeneration in the hyper-vulnerable environment of the AD brain, an effect mediated by microglial toxicity.

## Methods

### Animals

A colony of 5xFAD mice was initially established by mating C57Bl/6 J mice (originally supplied by Jackson) with heterozygote 5xFAD mice (5xFAD transgenic mouse model, carrying 5 mutated human genes associated with familial Alzheimer’s disease, APP K670N/M671L (Swedish), APP I716V (Florida), APP V717I (London), PS1 M146L, PS1 L286V), both a generous gift of Prof. Dani Frenkel from Tel Aviv University. Off springs of further generations were screened for carrying the transgenic cassettes (see below). To ascertain that 5xFAD and wild-type (wt) control mice had identical genetic background and environments, further breeding was performed by mating a heterozygote transgene-positive with a transgene-negative mouse, and all littermates were housed together. We made sure that each litter contained approximately 50% transgene-positive mice. Both male and female mice were used, maintaining equal distribution between experimental groups. Breeders were not used for experiments. Animal experimentation was approved by the institutional ethics committee, approval number MD-17-15057-4. All animal experimental groups were sized *n* = 5–7, unless stated otherwise.

### Mice genetic screening

Mice tails were sampled for polymerase chain reaction (PCR) analysis of APP, PS1, and a control mouse gene. DNA was extracted from mice tails using 50 mM Tris HCl (pH = 8), 0.05% Triton X-100, and 19.6 mg/mL Proteinase K. The vials were then heated to 55 °C for 15 min followed by 5 min in 80 °C. The PCR reaction mixture included 5 μL of DNA, 300 nM of the appropriate forward and reverse primers (Syntezza, Israel), and 5 μL of the master mix buffer containing nucleotides and Red Load Taq polymerase (Larova) in a total volume of 25 μL. Gene amplification was carried out using the GeneAmp 9700 Sequence Detection System (Applied Biosystems). For APP analysis, amplification included one stage of 3 min at 94 °C, followed by 35 cycles of a 3-step loop: 30 s at 94 °C, 1 min at 55 °C, and 1 min at 72 °C, followed by 2 min in 72 °C and cooling to 10 °C. For PS1 analysis, 35 cycles of a 3-step loop, 20 s at 94 °C, 1 min at 60 °C, and 1 min at 72 °C, followed by 2 min in 72 °C and cooling to 10 °C.

### ICV injections and surgical insertion of ICV pump

Mice were anesthetized using a combination of ketamine (80 mg/kg; i.p.) and xylazine (20 mg/kg; i.p.) prepared in normal saline. Single intracerebroventricular (ICV) injection of 15 μg, 25 μg, or 70 μg zymosan was performed using a stereotaxic device, at coordinates *A* = 0, *L* = 1, *H* = 2.5. For continuous delivery of zymosan, mice were implanted with Alzet pumps (1007D or 1004, according to manufacturer’s instructions, and as previously described [3] enabling constitutive 1-week or 28 days release of 25 μg zymosan. Mice were sacrificed either 24 h, 3, 14 or 28 days following ICV injection or after 7 or 28 days of ICV pump by perfusion. The selective TLR2 antagonist CU-CPT22 (Tocris Bioscience) was delivered ICV (at 10 μg/day) continuously for 2 weeks using implanted Alzet pumps (model 1002).

### IP injections of zymosan and lipoteichoic acid

Three repeated injections of zymosan (Z2843, BioParticles TexasRed conjugate, Invitrogen, 5 mg/animal/injection) or Purified LTA-SA (tlrl-pslta, Invivogen, 500 μg/animal/injection) were performed on days 0, 3, and 5. Animals were sacrificed on day 14 by perfusion.

### Histopathology

Animals were anesthetized with a lethal dose of pentobarbital, and the brains were perfused via the ascending aorta with ice-cold phosphate-buffered saline followed by cold 4% paraformaldehyde. Tissues were deep-frozen on dry ice, serial 10 μm coronal sections were prepared, and immune-fluorescent stainings were performed as previously described [1]. The following antibodies were used: mouse anti-NeuN (1:200, Chemicon), rabbit anti-glial fibrillary acidic protein (GFAP, 1:200, Dako), mouse anti-TLR2 (1:50, Abcam), rabbit anti-Iba1 (1:220, Wako) rat anti-CD31 (1:50, BD Pharmingen), mouse anti-nestin (1:200, Millipore), and mouse anti-p65 (for NfKb, 1:100, Santa Cruz SC-8008). Goat anti-rabbit Alexa-fluor 488 (1:200, Invitrogen), goat anti-rat Alexa-fluor 488 (1:200, Invitrogen), and goat anti-mouse Alexa-fluor 555 (1:200, Invitrogen) were used as secondary antibodies where appropriate.

### Quantification of cortical cell density

Each mouse brain was evaluated in coronal sections at the level of Bregma = 0.00 ± 0.1 mm and Bregma 1.0 ± 0.1 mm. The data are presented as average of counts in the two Bregmas. Two microscopic images of Dapi-stained nuclei/Neun/GFAP were obtained in a blinded manner from the left cortical hemisphere of each brain section (the contralateral side of pump-insertion/ICV injection insult) at × 20 magnifications (4 sections, 2 fields per section).

Computerized quantification of nuclei (DAPI) was performed using ImageJ (Process  Binary  Watershed  Analyze Particles), using a size-based threshold. An average value was calculated per animal, followed by calculation of the group (± SEM) average values.

To measure neuronal cell density in the cortex, sections were stained for NeuN and counted manually in a blinded manner adhering to stereological principles. Two adjacent × 20 fields midway between the pial lining and the corpus callosum at Bregma *L* = 1.0 mm were counted from the left hemisphere, contralateral to the side of injection (4 sections, 2 fields per section).

In order to evaluate the astrocytic density in the cortex, sections were stained for GFAP. Two microscopic images were obtained at the same position as for Neun and DAPI quantification, at a magnification of × 20 and identical camera exposure (4 sections, 2 fields per section). Computerized analysis was performed for the fraction of GFAP-stained area from total area of the image (*n* = 4). Both NeuN and GFAP were calculated as average values per mouse, followed by calculation of group average.

### BBB permeability evaluation and fluorescent zymosan injection

To analyze BBB permeability, 1 mg of 5-(and 6)-tetramethylrhodamine biocytin (Biocytin-TMR, T12921, Invitrogen) diluted in 100 μl of PBS was injected per mouse (*n* = 4 mice/group) intravenously into the tail vein. Seconds after the injection, the ears of injected mice turn pink, indicating the penetration of the material to blood circulation. Thirty minutes after injection, animals were sacrificed, and the brains were extracted and cut as described above. Slides were then stained with CD31 (557355, BD Pharmigen) and DAPI. For each mouse, 8 brain sections were screened (4 brain sections for each Bregma). Images were obtained using Nikon Confocal A1R. Fluorescent-conjugated zymosan A was injected IV to the tail vein followed by mice sacrifice 30 min later.

### Isolation and growth of adult brain microglia/macrophage

Microglia isolation was performed on 7-month-old 5xFAD and wt mice. Mice were terminally anesthetized and decapitated, and the brains were excised into Earl’s based salt solution. The brains were dissociated to a single-cell suspension using the Neural Tissue Dissociation Kit (P) (Miltenyi Biotec, 130–092–628) according to manufacturer’s protocol. For myelin removal, Percoll (GE Healthcare) protocol was performed. Microglia were isolated from myelin-free single-cell suspension using CD11b-conjugated beads (Miltenyi Biotec, 130-093-634) according to manufacturer’s protocol followed by depletion with MS columns (Miltenyi Biotec, 130–042–201). The cells were counted followed by either RNA extraction or seeded for in vitro ROS and NO measurements.

### RNA extraction and real-time PCR

Total RNA was isolated from freshly isolated microglia using RNA Mini Kit (Bioline) according to manufacturer’s instructions. cDNA was prepared from 100 ng of total RNA using qScript cDNA Synthesis Kit (Quantabio) according to manufacturer’s instructions. For real-time (RT) PCR, the reaction mixture included 1 μL of cDNA, 300 nmol/L concentrations of the appropriate forward and reverse primers (Syntezza, Israel), and 5 μL of SYBR green mix PerfeCTa SYBR Green FastMix Rox in a total volume of 10 μL. Gene amplification was carried out using the GeneAmp 7000 PCR system (Applied Biosystems). The gene expression results were normalized to the TBA gene. Three independent experiments were performed; each gene expression is evaluated in triplicates and is presented as mean ± SE. For the mRNA quantification, the fold change was normalized to the TBA transcript (ΔCT).

### FACS analysis

Microglia were isolated from the brains as described above. TLR2 expression was assessed immediately after the isolation using PE-anti-mouse CD282 (TLR2) (148603, Biolegend) by fluorescence-activated cell sorter (FACS) analysis (Beckman Coulter).

### ROS and NO measurement assays

5xFAD- and wt-isolated brain microglia were seeded on 96 well dark-sided plates covered with polylysine, at a density of 5 × 10^5^ cells per well with or without zymosan activation for 1 h (200 ng/ml). Cellular reactive oxygen species (ROS) were detected using DCFDA dye according to the manufacturer’s protocol (Abcam**,** AB-ab113851). Cell fluorescence was quantified using an ELISA plate reader (Beckman Coulter DTX 880 multimode detector). For the NO assay, brain microglia were isolated from 5xFAD and wt mice and seeded on 96 flat transparent plates (Nunc) covered with polylysine, at a density of 3 × 10^5^ cells per well with or without zymosan activation for 24 h (200 ng/ml). NO production was assessed using Greiss Reagent System according to the manufacturer’s protocol (Promega, G2930). Optical absorbance was quantified using an ELISA plate reader (Tecan Spark 10 M).

### Statistical analysis

The values are provided as mean ± SD or SE, as appropriate, and as described for each figure. The significance of quantifications was calculated using Student’s independent paired or unpaired samples *t* test as appropriate.

## Results

### Systemic TLR2 agonists induce neurodegeneration in 5xFAD brains by penetrating the CNS

**Fig. 1:**
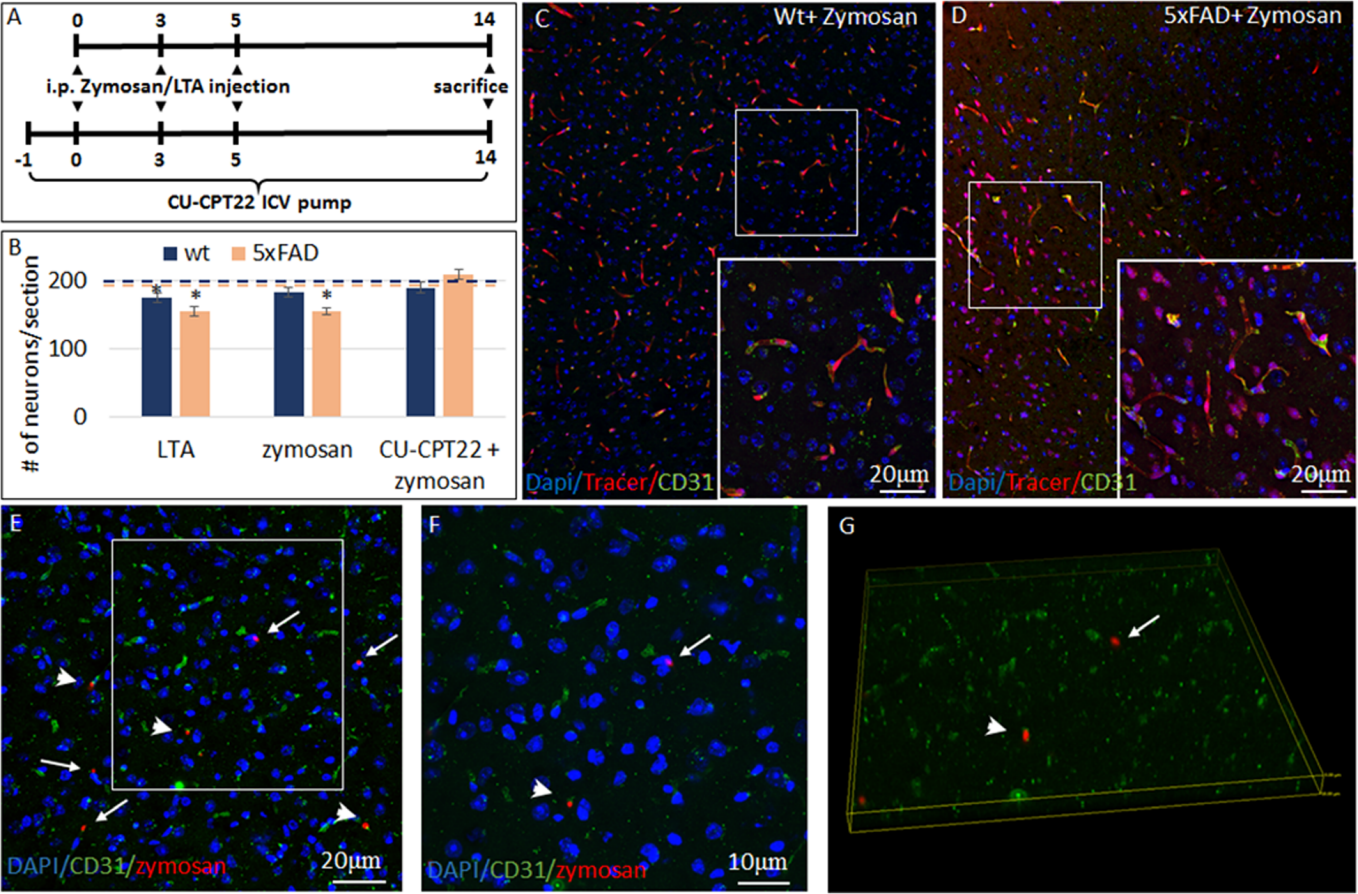
Microbial TLR2 agonist cross the blood-brain barrier and induce neurodegeneration after systemic delivery. **a** Zymosan or lipoteichoic acid were repeatedly injected intraperitoneally to naïve animals or to animals that have been implanted with an ICV pump continuously administering the TLR2 antagonist CU-CPT22. Mice were sacrificed at day 14 to assess neuronal loss. **b** Following 3 intraperitoneal injections of zymosan or lipoteichoic acid there was significant more loss in cortical neuronal counts in 5xFAD than in wt mice. ICV administration of TLR2 antagonist, CU-CPT22 abolished zymosan-induced neuronal loss. Dashed line represents the baseline NeuN count of untreated wt (blue) and 5xFAD (orange) mice. *Compared with untreated mice. **c** In wt mice that received intraperitoneal injections of zymosan, followed by intravenous injection of Biocytin-TMR, the BBB tracer was found exclusively within CD31+ blood vessels. **d** In 5xFAD, zymosan-injected mice Biocytin-TMR crossed the BBB effectively and accumulated in parenchymal brain cells. **e**–**g** In 5xFAD mice, pretreated with three intraperitoneal zymosan injections, a fourth injection of fluorescent-labeled zymosan resulted in multiple fluorescent foci in the brain parenchyma (enlarged **f** inserted in **e**). Arrowheads show a fluorescent zymosan particle associated with CD31+ blood vessels, arrows show a fluorescent zymosan particle that exited the blood vessel. 3-D confocal images confirmed the exiting of labeled zymosan from CD31+ blood vessels into the brain parenchyma (**g**)

Multiple types of common microorganisms express TLR2 agonists that may be released to the systemic circulation during infectious events. We examined whether systemic administration of two types of microbial wall-derived TLR2 agonists can cause loss of cortical neurons by quantifying NeuN + cells. Zymosan is a β-glucan polysaccharide TLR2 agonist derived from the yeast *Saccharomyces cerevisiae*. Since yeasts only rarely cause an infection in humans, we examined also the effect of lipoteichoic acid (LTA), a TLR2 agonist that is a major constituent of the bacterial wall in common gram-positive pathogens. Their neurotoxic effects were examined in wild-type and compared with 7-month-old 5xFAD mice, a time point when there is heavy amyloid deposition, microgliosis, and astrogliosis but no spontaneous loss of cortical neurons [10]. The TLR2 agonists were repeatedly administered by intraperitoneal injection for three times every other day (Fig. 1a, upper axis), in a sufficiently low dose, not to cause major systemic sickness. Systemically injected Zymosan induced a 3-fold larger loss in cortical neurons in 5xFAD mice, as compared with wt mice. There was 20 ± 4.3% neuronal loss in 5xFAD mice compared with untreated mice (*p* = 0.0003), versus 7.4 ± 3.5% loss in wt mice, compared with untreated mice (*p* = 0.06). This difference expressed a significantly increased vulnerability of 5xFAD mice as compared with wt mice (Fig. 1b, *p* = 0.005). LTA induced 11.5 ± 4.7% loss in cortical neurons in wt mice (*p* = 0.04 compared with untreated mice) and 20 ± 8.1% loss in 5xFAD mice (*p* = 0.005 compared with untreated mice), indicating a 2-fold larger loss in 5xFAD mice than wt mice (*p* = 0.08, Fig. 1b). Thus, systemic administration of low-dose microbial TLR2 agonists kills cortical neurons, and the 5xFAD brain shows increased susceptibility to their neurotoxic effect.

Systemic zymosan may activate various peripheral responses that affect the brain. In addition, zymosan is not completely TLR2-specific, activating an inflammatory response also via Dectin- 1[33]. To examine whether the systemic microbial TLR2 agonist effect was mediated through the brain TLR2, we continuously delivered intracerebroventricularly (ICV) CU-CPT22 (Fig. 1a, lower axis), a specific TLR2 antagonist, and then performed the repeated systemic injections of Zymosan. ICV CU-CPT22 abrogated completely the neurotoxic effect of Zymosan (Fig. 1b). Thus, systemic zymosan neurotoxicity is mediated by the brain TLR2, raising the possibility that it penetrates the CNS to induce neurodegeneration directly. We therefore examined blood-brain barrier integrity following systemic administration of the TLR2 agonist. After repeated systemic administration of low-dose Zymosan, intravenous injection of Biocytin-TMR showed little evidence of tracer signal in brain parenchyma outside blood vessels in wt mice (Fig. 1c). The disruption of the BBB in transgenic mouse models of AD is well established [24]. Indeed, in 5xFAD mice there was marked and consistent penetration of the tracer into the CNS parenchyma in all zymosan-pretreated mice (Fig. 1d). We therefore examined whether the TLR2 agonist itself penetrates the permeable BBB of 5xFAD mice. Fluorescent-labeled zymosan was administered systemically, after three previous systemic injections of unlabeled zymosan. The fluorescent-labeled zymosan was found both within blood vessels and in the CNS parenchyma of 5xFAD mice (Fig. 1e–g). In sum, the 5xFAD brain, at a time point of advanced AD pathology (but prior to neurodegeneration), is significantly more vulnerable to the neurotoxicity of the TLR2 agonist than wild-type brains. BBB disruption in 5xFAD mice enables the penetration of TLR2 agonists to the CNS parenchyma, to kill cortical neurons.

### Neurodegeneration by microbial TLR2 agonist may be due to repeated hits

**Fig. 2:**
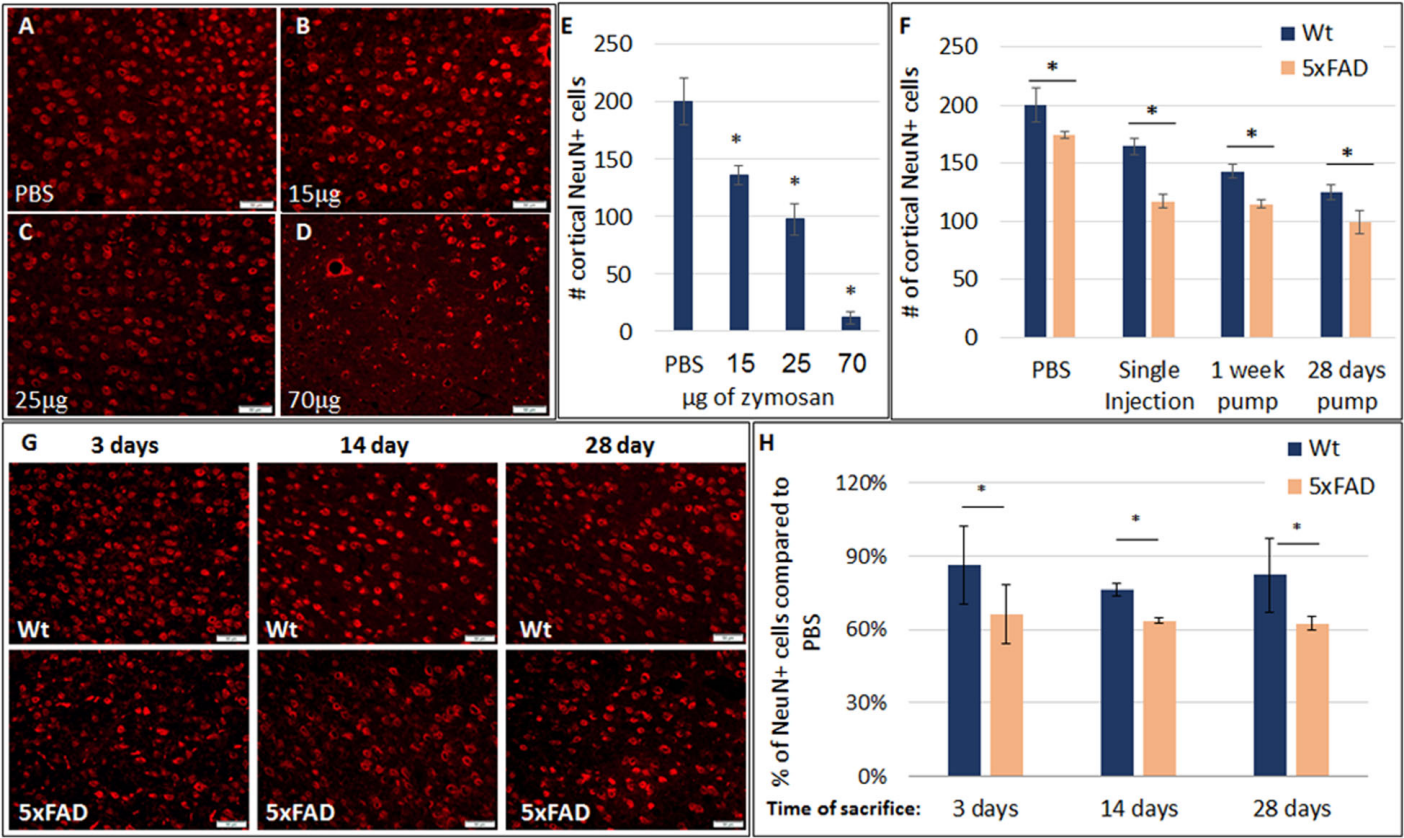
Increased susceptibility of 5xFAD brains to the neurotoxic effect of zymosan. **a**–**e** Zymosan was delivered ICV continuously for 28 days in wt mice, and cortical neurons were then quantified. Panels **a**–**d** show representative images of the cortex stained for Neun+ neurons injected with PBS (**a**), 15 μg (**b**), 25 μg (**c**) or 70 μg (**d**) zymosan. Quantification indicated a dose-dependent neurotoxic effect of zymosan, inducing neurodegeneration (**e**). **f** Twenty-five micrograms zymosan was delivered ICV as single injection, or continuously for 1 week or 28 days in wt and 5xFAD mice. The extent of cortical neuronal loss was dependent on time of exposure. At all durations of exposure, 5xFAD brains exhibited a significantly increased loss of cortical neurons as compared with wt mice. **g**, **h** The dynamics of neuronal loss was examined at 3 days, 2 weeks and 28 days after a single ICV injection of zymosan. Panels in **g** show representative images of the cortex stained for Neun+ neurons in wt and 5xFAD brains at the different time points. Quantification of cortical neuronal density showed that zymosan-induced neuronal death within 3 days after injection (**h**)

To further elucidate the mechanisms by which microbial TLR2 agonists induce neurodegeneration and to avoid potential systemic effects, we performed further experiments by delivering zymosan ICV directly. First, a dose-response experiment was performed in 7-month-old wild-type C57Bl/6 mice (*n* = 3/group) by continuous ICV delivery of zymosan for 28 days. There was a dose-dependent loss of cortical neurons (Fig. 2a–e). To determine the time of exposure effect, further experiments were performed using a medium dose of Zymosan. A total dose of 25 μg zymosan was delivered ICV either by a single injection or by 1-week or by 28-days continuous release, and NeuN+ cortical neurons were quantified after 1 month. In wt mice, there was time-of-exposure dependent neuronal loss (Fig. 2f, *f* (3, 17) = 17.963 *p* < 0.001 ANOVA). 5xFAD mice exhibited significantly increased vulnerability to the neurotoxic effect of zymosan, with significantly increased neuronal loss on all durations of exposure (Fig. 2f). The difference was most prominent after a single injection of zymosan, which induced a 2.7-fold larger loss of cortical neurons in 5xFAD as compared with wt mice (35% versus 13%, *p* = 0.03). These further highlights the susceptibility of the brains inflicted with AD pathology to even short-term exposures to the TLR2 agonist. We then examined whether the TLR2 agonist induces acute neuronal death or triggers a progressive neurodegenerative process. To that end, a single ICV injection of 25 μg zymosan was performed and the 5xFAD mice were sacrificed at 3 days, 2 weeks and 4 weeks post-injection for quantification of cortical neurons. The full neurotoxic effect of zymosan was evident already at 3 days after injection, indicating an acute neurotoxic effect (Fig. 2g–h). Thus, the 5xFAD brain exhibits consistent increased vulnerability to the TLR2 agonist, across multiple doses and exposure times. The acute neurotoxic effect of zymosan suggests that it does not trigger an independent neurodegenerative process, but rather repeated exposures may result in accumulated loss of brain neurons.

### Neurotoxicity of TLR2 agonist is selective for neurons and is mediated by microglia

**Fig. 3:**
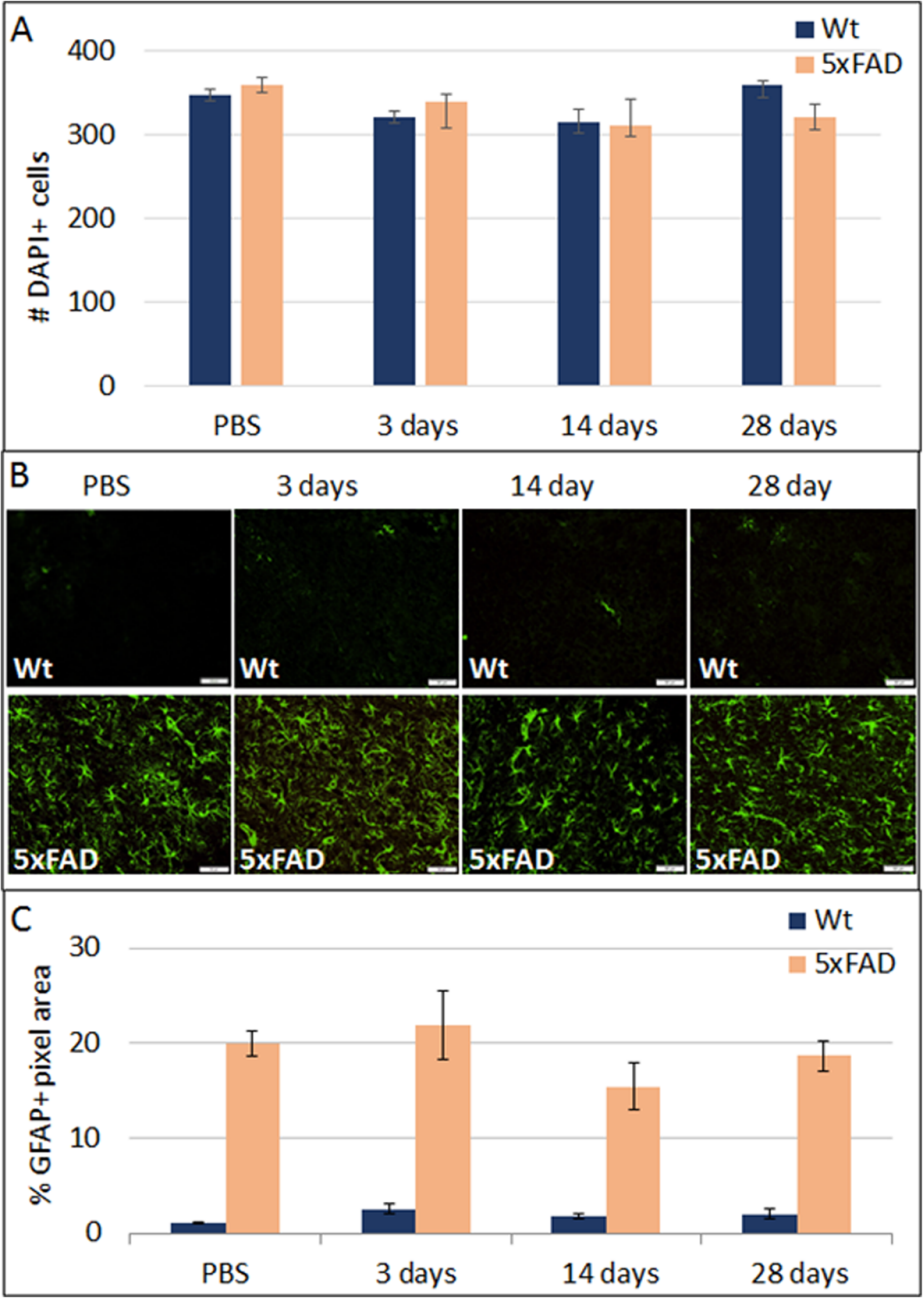
Zymosan neurotoxicity is selective for neurons. **a** Quantification of total cortical cell number was performed by counting Dapi+ nuclei. Total cell numbers were not affected by ICV injection of zymosan. **b** Staining for GFAP+ astrocytes showed ICV injection of zymosan had a minimal effect in wt mice at 3 days, 2 weeks, and 28 days as compared with PBS-injected mice. In the 5xFAD brains, there was strong GFAP immune-reactivity in PBS-injected mice, which did not further increase in response to zymosan ICV. **c** Quantification of GFAP-immune-reactivity (as % of image area) showed the massive astrogliosis in 5xFAD mice at all time points, unaffected by zymosan

We then examined whether the TLR2 agonist neurotoxicity is selective for neurons. The total number of cortical cells and GFAP immunoreactivity were quantified in adjacent fields to the NeuN+ neuron quantification in the zymosan-injected brains. While there was significant neuronal loss, zymosan did not induce any significant change in the total number of cells (Fig. 3a). The basal GFAP immune-reactivity in 5xFAD mice was very strong as compared with wild-type mice, in agreement with the previous reports [10, 27]. Zymosan did not induce significant change in GFAP immune-reactivity in wt and 5xFAD mice (Fig. 3b–c). These findings indicate a neuron-selective killing effect by zymosan.

**Fig. 4:**
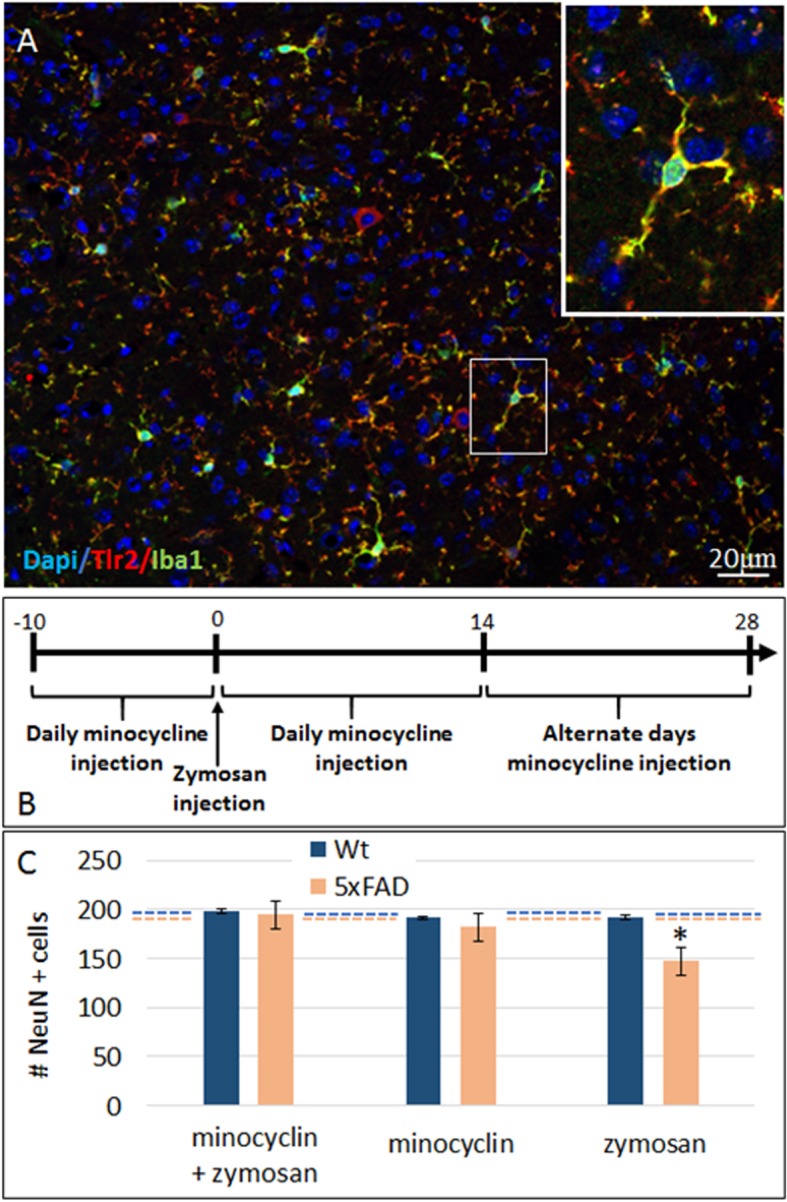
Zymosan-induced neurotoxicity is mediated by the brain microglia. **a** Staining of cortical sections from 5xFAD brain showed that TLR2 immune-reactivity co-localized with Iba1+ microglia. There were rare cells with neuron-like morphology that also expressed TLR2. **b** Experimental time line of minocycline treatment and zymosan injection. **c** Quantification of cortical neurons showed that minocycline treatment did not affect neuronal counts, but abolished the neurotoxic effect induced by ICV injection of zymosan. Dashed line represents the baseline NeuN count of untreated wt (blue) and 5xFAD (orange) mice. *ANOVA *f* (3, 20) = 22.093, *p* < 0.001

Cumulative data suggests that neurodegeneration in AD is driven by the neurotoxicity of activated microglia [36]. We therefore examined whether zymosan-induced neuronal loss is caused by a direct effect on neurons or mediated by activated resident microglia. First, TLR2 expression was examined in the brain of 7-month-old wt and 5xFAD mice. TLR2 immune-reactivity co-localized with IBA-1+ microglia (Fig. 4a). To examine whether the neurotoxic effect of the TLR2 agonist is mediated by microglia, we treated mice with minocycline, a microglial inhibitor. First, to verify the immunosuppressive effect of minocycline, the density of IBA1+ microglia was quantified in zymosan-treated and untreated wild-type mice after Minocyclin treatment. Daily intraperitoneal (i.p.) injections of 0.35 mg minocycline/10 g weight were performed for 38 days. After 14 days of minocycline treatment, one group of animals was injected ICV with 25 μg zymosan. Minocycline caused a 50 ± 6.3% reduction of IBA1+ cell counts in naïve mice (*p* = 0.005), as well as 37 ± 4.6% reduction in zymosan-treated mice (*p* = 0.007, data not shown). Then, we examined zymosan-induced neuronal loss in groups of wild-type and 5xFAD mice that were treated with minocycline as described (Fig. 3b). Minocycline alone did not affect neuronal counts at this time point, which is prior to neurodegeneration in 5xFAD mice (Fig. 4c). In minocycline-treated 5xFAD mice the neurotoxic effect of zymosan was abolished (Fig. 4c). Thus, TLR2-mediated neurodegeneration is mediated by resident brain microglia.

### Increased TLR2 expression and microglial neurotoxicity in 5xFAD mice brains

**Fig. 5:**
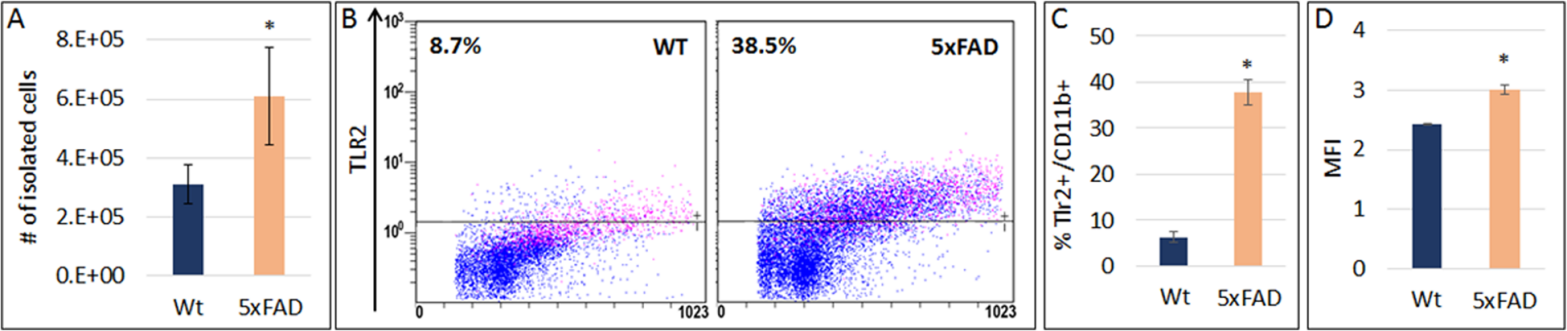
5xFAD cortex contains higher number of TLR2+ microglia. **a** A 2-fold higher amount of CD11b + microglia was isolated from the cortex of 5xFAD as compared with wt mice. **b**–**d** FACS analysis of TLR2 expression in isolated CD11b + microglia from wt and 5xFAD mice. There was a 6-fold increase in the fraction of TLR2-expressing microglia in 5xFAD mice (**b**, **c**, *p = 0.0002). Mean fluorescence intensity (MFI) was 20% higher in TLR2-expressing microglia from 5xFAD as compared with wt mice (**d**, *p = 0.0006)

AD pathology is characterized by an increase in the number and in the activated phenotype of resident microglia [16]. In agreement, the 5xFAD mouse brain exhibited increased microglial density. The number of brain cortex CD11B+ microglia in 7-month-old 5xFAD was almost 2-fold higher as compared with age-matched wild-type mice (Fig. 5a, *p* = 0.015). FACS analysis showed a 6-fold increase in the fraction of TLR2+ microglia from 5xFAD brains as compared with wt mice (Fig. 5b–c). Evaluation of TLR2 immune-reactivity in the fraction of TLR2+, CD11b + cells by mean fluorescence intensity showed that in microglia isolated from zymosan-treated 5xFAD mice there was 20% higher TLR2 expression, as compared with wild-type microglia (Fig. 5d). These findings suggest that the increased vulnerability of 5xFAD mice to zymosan-induced neurotoxicity may be mediated partly by an increase in the basal density of microglia, as well as by an increase in the fraction of TLR2+ microglia in their brains.

**Fig. 6:**
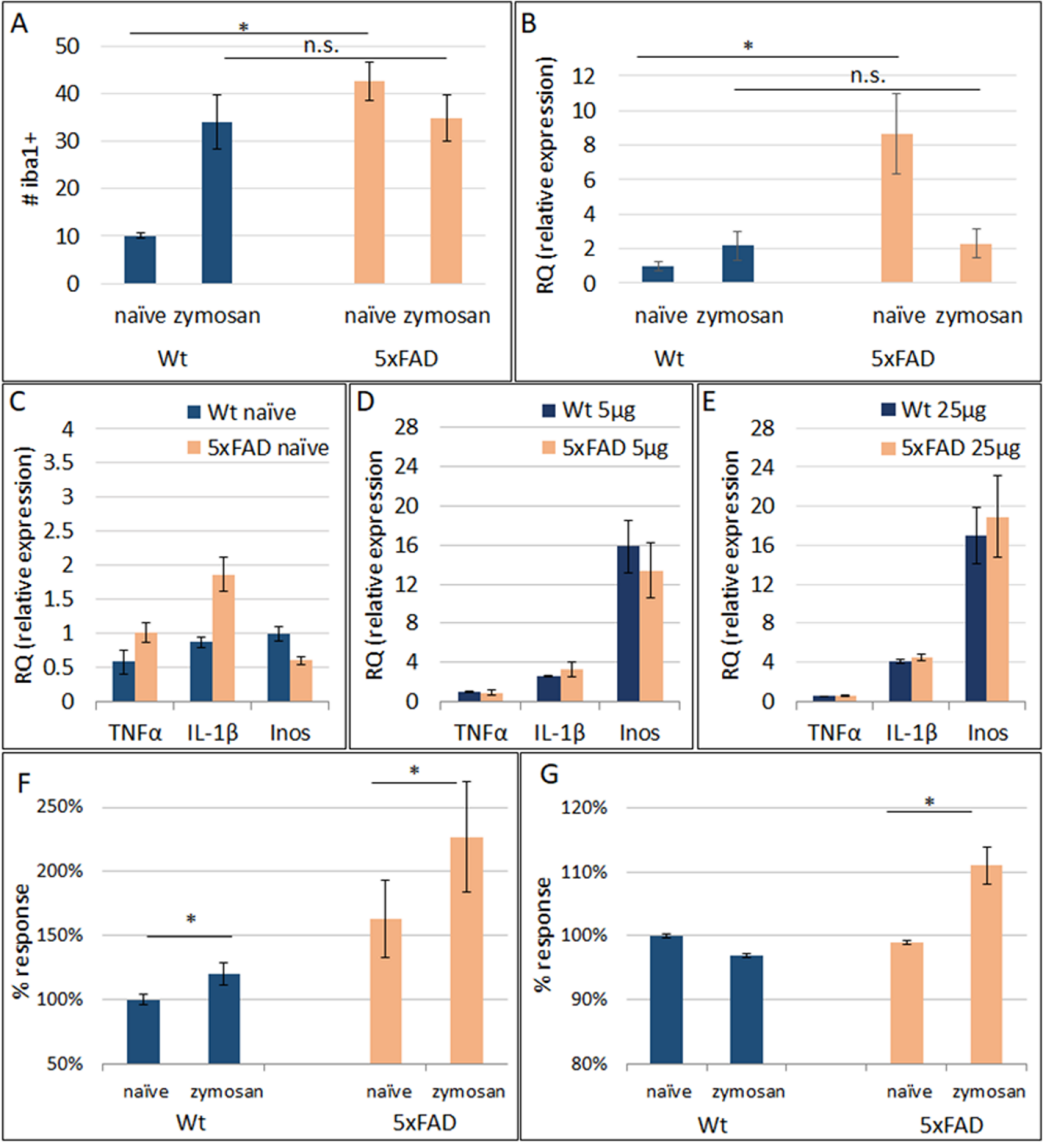
TLR2 agonist increases microglial neurotoxic phenotype, but not inflammatory cytokines in 5xFAD microglia. **a** IBA1+ cell counts in cortical sections showed a basal 4-fold higher number of microglia in 5xFAD mice. Zymosan induced a marked increase in microglial number in wt mice but not in 5xFAD mice. **b** Real-time PCR for TLR2 expression in isolated CD11b + cells showed an 8-fold higher basal expression. Following TLR2 agonist stimulation there was 2-fold increased expression in wt, but 4-fold decreased TLR2 expression in 5xFAD microglia. **c** Real-time PCR showed mild differences in basal expression of inflammatory cytokines. **d**, **e** ICV injection of both low (5 μg, **d**) and medium dose (25 μg, **e**) zymosan induced similar cytokine expression in wt and 5xFAD microglia. **f** In vitro stimulation of microglia with zymosan caused significantly larger increase in ROS production in 5xFAD than in wt microglia. **g** In vitro stimulation of microglia with Zymosan caused a significant increase in NO production in 5xFAD but not in wt microglia

In view of the differences in the basal microglial state in 5xFAD versus wt mice, it was important also to assess microglial density and function in response to stimulation with the TLR2 agonist. Injection of Zymosan induced a marked increase in IBA1+ cell density in the wild-type brains to a comparable value of untreated 5xFAD brains (Fig. 6a). Zymosan did not induce any further increase in microglial density in 5xFAD mice (Fig. 6a). This indicates that the AD pathology-induced increase in microglial density could not be further enhanced by exposure to the TLR2 agonist. We therefore examined whether there are also functional differences that underlie the increased TLR2-mediated neurotoxicity of microglia in the 5xFAD brains. We examined the level of TLR2 gene and protein expression in CD11b + microglia that were isolated from naïve or 24 h after ICV injection of 25 μg zymosan in 7-month-old 5xFAD versus wild-type mice brains. RT-PCR in microglia from wt mice showed that zymosan injection induced a 2-fold increase in TLR2 mRNA expression. In 5xFAD microglia the basal TLR2 mRNA expression was 8-fold higher than in wild-type microglia, but was reduced following Zymosan injection to a comparable level as in wild-type zymosan-treated microglia (Fig. 6b). We examined microglial cytokine response at 24 h after ICV zymosan injection. Basal levels of IL-1β and TNFα were 2-fold higher in 5xFAD microglia as compared with wild type (Fig. 6c). However, both low and medium doses of zymosan induced similar increases in the expression of these genes in wt and 5xFAD-derived microglia (Fig. 6d–e), indicating that microglia from 5xFAD mice did not exhibit increased cytokine response to minute doses of zymosan. We therefore examined microglial neurotoxic phenotype as determined by the actual production of reactive oxygen species (ROS) and nitric oxide (NO). First, we confirmed that TLR2 activation of isolated CD11b + microglia by zymosan induces nuclear translocation of *NfKb*, as a well described downstream pathway of TLR2-mediated immune activation (data not shown). Then, ROS and NO production were quantified in freshly isolated microglia from 7-month-old wild-type and 5xFAD mice, following in vitro stimulation with zymosan. Basal production of ROS was 1.62-fold higher in 5xFAD versus wild-type microglia (Fig. 6f). Zymosan induced a mild 18% increase in ROS production in wild-type microglia. In 5xFAD microglia, zymosan induced a significantly larger 40% increase in ROS production (*p* = 0.01) and was almost 2-fold higher than in zymosan-stimulated wild-type microglia (Fig. 6f, *p* = 0.05). Also, zymosan induced a significant increase in NO production by microglia from 5xFAD mice (*p* = 0.01) but not by microglia from wild-type mice (Fig. 6g).

In conclusion, the increased vulnerability of 5xFAD brains to the microbial TLR2 agonist is a result of higher basal density of TLR2+ microglia, as well as augmented neurotoxicity in response to the TLR2 agonist.

## Discussion

We show here that microbial wall-derived TLR2 agonists induce microglial neurotoxicity to kill cortical neurons. We found that AD brain pathology confers significantly increased vulnerability to microbial TLR2 agonists’ neurotoxicity through an increase in number and activation of TLR2+ microglia. Furthermore, we demonstrated that the AD pathology enabled penetration of systemically administered microbial TLR2 agonists to the CNS. It has been shown that systemic infections are associated with cognitive decline. Our findings further suggest that systemic infections may not only cause cognitive difficulties due to inflammatory cytokines-mediated sickness behavior [4], but may actually facilitate neurodegeneration. We propose that repeated insults by exposure to microbial TLR2 agonists during recurrent and chronic infections may result in accumulation of neuronal loss, facilitating the neurodegenerative process and cognitive deterioration.

Susceptibility of AD pathology-primed brains may occur in response to both CNS-derived TLR2 agonists and systemic pathogens. Herpes simplex virus derived glycoproteins B, H, and L, are TLR2 agonists [19]. Indeed, recent studies suggested a strong link between CNS infection with HSV and development of AD in human patients [14] and mice [5]. More commonly, TLR2 agonists are produced by multiple systemic infectious agents affecting patients, including chronic gingivitis [17], skin pathogens, [38] and gut microbiome [47]. For systemic infectious agents’ derived TLR2 agonists to cause neurodegeneration, a central issue is whether they cross the BBB to induce their neurotoxic effect directly. It has been suggested that repeated exposure to zymosan may disrupt the BBB [25]. Also, lipoteichoic acid (LTA), a TLR2 agonist that is a major constituent of the bacterial wall in common gram-positive pathogens, has been shown to disrupt the BBB [2]. Specifically, LTA disrupted BBB integrity through the activation of glial cells and induction of pro-inflammatory cytokines and of nitric oxide [2]. Systemic delivery of LTA downregulated the expression of the tight junction-associated proteins claudin 5 and occludin in the brain [22]. Indeed, LTA was shown to mediate *Staphylococcus aureus* bacterial neuro-invasion via the BBB [8, 34]. The notion that systemic TLR2 agonists may penetrate the CNS is especially attractive given the vast literature on BBB-breakdown in AD [39] and its animal models [24, 41, 44]. It has been suggested that BBB integrity is compromised in transgenic AD mice very early, even prior to amyloid deposition [44]. Moreover, it was shown that transgenic AD mice exhibited increased susceptibility to BBB disruption following induction of peripheral inflammatory states [41]. Finally, there are also multiple indications for BBB disruption early in the course of human AD [26, 40]. Our findings are in close agreement with previous literature. Moreover, we show that systemically injected TLR2 agonist zymosan penetrates the 5xFAD mouse brain parenchyma. Thus, release of microbial TLR2 agonists during peripheral infections may lead to their entering the CNS to activate directly local microglia and induce neurotoxicity.

The acquired susceptibility to neurotoxic stimulation of AD brain-derived microglia highlights the need to characterize their phenotype in AD. Both toxic activation and dysfunction of microglia play a role in propagation of neurodegeneration [32, 35]. AD pathology renders microglia both defective in their ability to engulf and remove Aβ from the brain (leading to further amyloid deposition and neurotoxicity) [13, 46] as well as to neurotoxic activation, affecting brain neurons [36]. We therefore compared the functional state of microglia from 5xFAD and wt mice, in terms of their TLR2-mediated neurotoxicity. We found that the AD pathology is associated with an increase in TLR2+ microglia in the brain, which produce increased amounts of ROS and NO in response to stimulation with a TLR2 agonist, leading to neuronal death. These findings underscore the pivotal role of TLR2 in mediating neurodegeneration in AD and may therefore represent an important therapeutic target [30].

Aβ has been shown to bind to and activate TLR2 and can therefore trigger microglial neurotoxicity [31]. Furthermore, TLR2 seems pivotal for determining the neurotoxic versus neuroprotective profile of microglia [45]. However, a gap of over 15 years may pass before the brain that is burdened with marked Aβ deposition displays significant atrophy [7]. In addition, pathological studies support the notion that Aβ pathology is not associated directly with neurodegeneration. While the toxic effect of other abnormal misfolded proteins, such as phosphorylated Tau and TDP43, superimposed on Aβ pathology has been suggested [12, 42], environmental factors and specifically infectious agents may facilitate as well the neurodegenerative process in the brain that has been primed by the Aβ pathology [18]. Thus, we propose that the common basic pathologic feature of amyloid deposition that is shared by all Alzheimer’s disease patients is associated with increased susceptibility to various internal and external insults. The course of disease in the individual patient may then depend on the type and extent of such insults, and in particular the extent of infections that the patient is exposed to. This notion underscores the importance of prevention medicine to maintain brain’s health in the aging, at-risk population.

## Conclusions

We propose a model representing the role of microbial wall-derived TLR2 agonists in AD pathogenesis. AD pathology involves microglial activation and dysfunction, and defective removal of Aβ may cause a vicious cycle increasing disease pathology and further microglial activation. Aβ-induced microglial activation may be insufficient to induce widespread neurodegeneration in itself. However, we propose that it confers increased susceptibility to systemic infections-induced neuronal death, mediated by TLR2 signaling. Repeated systemic microbial insults may therefore facilitate the neurodegenerative process in AD.

## Data Availability

No supporting data besides presented in the manuscript

## Abbreviations

AD: Alzheimer’s disease
CNS: Central nervous system
ICV: Intracerebroventricular
LTA: Lipoteichoic acid
PAMPs: Pathogen-associated molecular patterns
ROS: Reactive oxygen species
TLR2: Toll-like receptor 2

## References

[1] Ben-Hur T, Einstein O, Mizrachi-Kol R, Ben-Menachem O, Reinhartz E, Karussis D (2003). Transplanted multipotential neural precursor cells migrate into the inflamed white matter in response to experimental autoimmune encephalomyelitis. Glia.

[2] Boveri M, Kinsner A, Berezowski V, Lenfant AM, Draing C, Cecchelli R (2006). Highly purified lipoteichoic acid from gram-positive bacteria induces in vitro blood-brain barrier disruption through glia activation: role of pro-inflammatory cytokines and nitric oxide. Neuroscience.

[3] Cohen ME, Fainstein N, Lavon I, Ben-Hur T (2014). Signaling through three chemokine receptors triggers the migration of transplanted neural precursor cells in a model of multiple sclerosis. Stem Cell Res.

[4] Dantzer R (2001). Cytokine-induced sickness behavior: mechanisms and implications. Ann N Y Acad Sci.

[5] De Chiara G, Piacentini R, Fabiani M, Mastrodonato A, Marcocci ME, Limongi D (2019). Recurrent herpes simplex virus-1 infection induces hallmarks of neurodegeneration and cognitive deficits in mice. PLoS Pathog.

[6] De Strooper B, Karran E (2016). The cellular phase of Alzheimer’s disease. Cell.

[7] Donohue MC, Sperling RA, Petersen R, Sun CK, Weiner MW, Aisen PS (2017). Association between elevated brain amyloid and subsequent cognitive decline among cognitively normal persons. Jama.

[8] Doran KS, Engelson EJ, Khosravi A, Maisey HC, Fedtke I, Equils O (2005). Blood-brain barrier invasion by group B *Streptococcus* depends upon proper cell-surface anchoring of lipoteichoic acid. J Clin Invest.

[9] Dunn N, Mullee M, Perry VH, Holmes C (2005). Association between dementia and infectious disease: evidence from a case-control study. Alzheimer Dis Assoc Disord.

[10] Fainstein N, Dan-Goor N, Ben-Hur T (2018). Resident brain neural precursor cells develop age-dependent loss of therapeutic functions in Alzheimer’s mice. Neurobiol Aging.

[11] Fulop T, Itzhaki RF, Balin BJ, Miklossy J, Barron AE (2018). Role of microbes in the development of Alzheimer’s disease: state of the art - an International Symposium Presented at the 2017 IAGG Congress in San Francisco. Front Genet.

[12] Giannakopoulos P, Herrmann FR, Bussiere T, Bouras C, Kovari E, Perl DP (2003). Tangle and neuron numbers, but not amyloid load, predict cognitive status in Alzheimer’s disease. Neurology.

[13] Guillot-Sestier MV, Doty KR, Town T (2015). Innate immunity fights Alzheimer’s disease. Trends Neurosci.

[14] Harris SA, Harris EA (2015). Herpes simplex virus type 1 and other pathogens are key causative factors in sporadic Alzheimer’s disease. J Alzheimer’s Dis.

[15] Holmes C, El-Okl M, Williams AL, Cunningham C, Wilcockson D, Perry VH (2003). Systemic infection, interleukin 1beta, and cognitive decline in Alzheimer’s disease. J Neurol Neurosurg Psychiatry.

[16] Hopperton KE, Mohammad D, Trepanier MO, Giuliano V, Bazinet RP (2018). Markers of microglia in post-mortem brain samples from patients with Alzheimer’s disease: a systematic review. Mol Psychiatry.

[17] Jain S, Coats SR, Chang AM, Darveau RP (2013). A novel class of lipoprotein lipase-sensitive molecules mediates toll-like receptor 2 activation by Porphyromonas gingivalis. Infect Immun.

[18] Kamer AR, Fortea JO, Videla S, Mayoral A, Janal M, Carmona-Iragui M (2016). Periodontal disease’s contribution to Alzheimer’s disease progression in Down syndrome. Alzheimers Dement.

[19] Leoni V, Gianni T, Salvioli S, Campadelli-Fiume G (2012). Herpes simplex virus glycoproteins gH/gL and gB bind Toll-like receptor 2, and soluble gH/gL is sufficient to activate NF-kappaB. J Virol.

[20] Liu B, Hong JS (2003). Role of microglia in inflammation-mediated neurodegenerative diseases: mechanisms and strategies for therapeutic intervention. J Pharmacol Exp Ther.

[21] Liu S, Liu Y, Hao W, Wolf L, Kiliaan AJ, Penke B (2012). TLR2 is a primary receptor for Alzheimer’s amyloid beta peptide to trigger neuroinflammatory activation. J Immunol.

[22] Mayerhofer R, Frohlich EE, Reichmann F, Farzi A, Kogelnik N, Frohlich E (2017). Diverse action of lipoteichoic acid and lipopolysaccharide on neuroinflammation, blood-brain barrier disruption, and anxiety in mice. Brain Behav Immun.

[23] McDonald CL, Hennessy E, Rubio-Araiz A, Keogh B, McCormack W, McGuirk P (2016). Inhibiting TLR2 activation attenuates amyloid accumulation and glial activation in a mouse model of Alzheimer’s disease. Brain Behav Immun.

[24] Montagne A, Zhao Z, Zlokovic BV (2017). Alzheimer’s disease: a matter of blood-brain barrier dysfunction?. J Exp Med.

[25] Nagyoszi P, Wilhelm I, Farkas AE, Fazakas C, Dung NT, Hasko J (2010). Expression and regulation of toll-like receptors in cerebral endothelial cells. Neurochem Int.

[26] Nation DA, Sweeney MD, Montagne A, Sagare AP, D’Orazio LM, Pachicano M (2019). Blood-brain barrier breakdown is an early biomarker of human cognitive dysfunction. Nat Med.

[27] Oakley H, Cole SL, Logan S, Maus E, Shao P, Craft J (2006). Intraneuronal beta-amyloid aggregates, neurodegeneration, and neuron loss in transgenic mice with five familial Alzheimer’s disease mutations: potential factors in amyloid plaque formation. J Neurosci.

[28] Perry VH, Cunningham C, Holmes C (2007). Systemic infections and inflammation affect chronic neurodegeneration. Nat Rev Immunol.

[29] Perry VH, Newman TA, Cunningham C (2003). The impact of systemic infection on the progression of neurodegenerative disease. Nat Rev Neurosci.

[30] Rangasamy SB, Jana M, Roy A, Corbett GT, Kundu M, Chandra S (2018). Selective disruption of TLR2-MyD88 interaction inhibits inflammation and attenuates Alzheimer’s pathology. J Clin Invest.

[31] Richard KL, Filali M, Prefontaine P, Rivest S (2008). Toll-like receptor 2 acts as a natural innate immune receptor to clear amyloid beta 1-42 and delay the cognitive decline in a mouse model of Alzheimer’s disease. J Neurosci.

[32] Sarlus H, Heneka MT (2017). Microglia in Alzheimer’s disease. J Clin Invest.

[33] Shah VB, Huang Y, Keshwara R, Ozment-Skelton T, Williams DL, Keshvara L (2008). Beta-glucan activates microglia without inducing cytokine production in dectin-1-dependent manner. J Immunol.

[34] Sheen TR, Ebrahimi CM, Hiemstra IH, Barlow SB, Peschel A, Doran KS (2010). Penetration of the blood-brain barrier by *Staphylococcus aureus*: contribution of membrane-anchored lipoteichoic acid. J Mol Med.

[35] Shi Y, Holtzman DM (2018). Interplay between innate immunity and Alzheimer disease: APOE and TREM2 in the spotlight. Nat Rev Immunol.

[36] Spangenberg EE, Green KN (2017). Inflammation in Alzheimer’s disease: lessons learned from microglia-depletion models. Brain Behav Immun.

[37] Spangenberg EE, Lee RJ, Najafi AR, Rice RA, Elmore MR, Blurton-Jones M (2016). Eliminating microglia in Alzheimer’s mice prevents neuronal loss without modulating amyloid-beta pathology. Brain.

[38] Strunk T, Power Coombs MR, Currie AJ, Richmond P, Golenbock DT, Stoler-Barak L (2010). TLR2 mediates recognition of live Staphylococcus epidermidis and clearance of bacteremia. PLoS One.

[39] Sweeney MD, Montagne A, Sagare AP, Nation DA, Schneider LS, Chui HC (2019). Vascular dysfunction-the disregarded partner of Alzheimer’s disease. Alzheimer's Dementia.

[40] Sweeney MD, Sagare AP, Zlokovic BV (2018). Blood-brain barrier breakdown in Alzheimer disease and other neurodegenerative disorders. Nat Rev Neurol.

[41] Takeda S, Sato N, Ikimura K, Nishino H, Rakugi H, Morishita R (2013). Increased blood-brain barrier vulnerability to systemic inflammation in an Alzheimer disease mouse model. Neurobiol Aging.

[42] Thal DR, Del Tredici K, Ludolph AC, Hoozemans JJ, Rozemuller AJ, Braak H (2011). Stages of granulovacuolar degeneration: their relation to Alzheimer’s disease and chronic stress response. Acta Neuropathol.

[43] Tremlett H, Bauer KC, Appel-Cresswell S, Finlay BB, Waubant E (2017). The gut microbiome in human neurological disease: a review. Ann Neurol.

[44] Ujiie M, Dickstein DL, Carlow DA, Jefferies WA (2003). Blood-brain barrier permeability precedes senile plaque formation in an Alzheimer disease model. Microcirculation.

[45] Wang J, Zhao D, Pan B, Fu Y, Shi F, Kouadir M (2015). Toll-like receptor 2 deficiency shifts PrP106-126-induced microglial activation from a neurotoxic to a neuroprotective phenotype. J Mol Neurosci.

[46] Wang S, Colonna M (2019). Microglia in Alzheimer’s disease: a target for immunotherapy. J Leukoc Biol.

[47] Zhang D, Chen G, Manwani D, Mortha A, Xu C, Faith JJ (2015). Neutrophil ageing is regulated by the microbiome. Nature.

